# Simultaneous Leadless Pacemaker and Subcutaneous ICD Implantation With Intraoperative Screening: Workflow in Two Patients

**DOI:** 10.1016/j.jaccas.2022.06.018

**Published:** 2022-11-03

**Authors:** Jonathan Nieves, David B. Laslett, Anuj Basil, Isaac R. Whitman, Joshua M. Cooper, Edmond M. Cronin

**Affiliations:** Lewis Katz School of Medicine at Temple University, Philadelphia, Pennsylvania, USA

**Keywords:** bradycardia, cardiac pacemaker, cardiomyopathy, cardioversion, primary prevention, secondary prevention, ventricular fibrillation, ventricular tachycardia, CIED, cardiovascular implantable electronic device, ICD, implantable cardioverter-defibrillator, LP, leadless pacemaker, LVAD, left ventricular assist device, S-ICD, subcutaneous implantable cardioverter-defibrillator, VF, ventricular fibrillation

## Abstract

A communicating subcutaneous implantable cardioverter-defibrillator (ICD) and leadless pacemaker system is being developed for patients who require both pacing and ICD therapy. It is important to ensure that the paced morphology from the leadless pacemaker will be sensed appropriately by the subcutaneous ICD. We present 2 cases illustrating our approach and workflow. (**Level of Difficulty: Intermediate.**)

## Central Illustration

**Figure:**
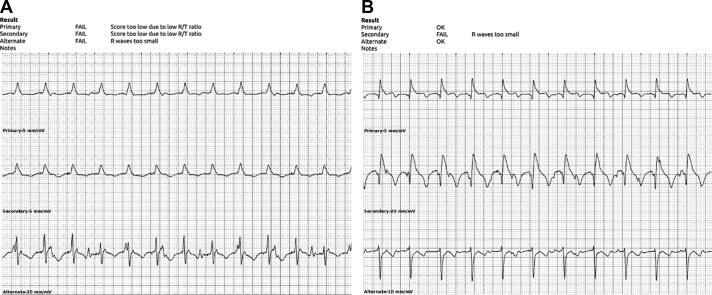

Cardiovascular implantable electronic device (CIED) infections have increased in the past several decades due to expanding device indications and the need for device upgrades and generator changes.^1^ Both subcutaneous implantable cardioverter-defibrillators (S-ICD) and leadless pacemakers (LP) have emerged as options for patients requiring ICD and pacing therapy, respectively, including those with prior CIED infection, particularly if the prior infection was lead-associated endocarditis.^2^^,^^3^ However, many patients benefit from both pacing and tachycardia therapy capability, and therefore either S-ICD or LP separately would be insufficient.^4^, ^5^, ^6^, ^7^, ^8^, ^9^, ^10^, ^11^, ^12^, ^13^, ^14^, ^15^ The following 2 cases illustrate our approach to implantation of this combination system to avoid double-counting by the S-ICD during ventricular pacing, as well as avoid undersensing of ventricular tachyarrhythmias if ventricular pacing inappropriately continued from the LP.

**Learning Objectives**

• To describe a workflow for simultaneous implantation of a leadless pacemaker and a subcutaneous ICD that ensures satisfactory sensing of paced R waves.
• To review the potential interactions between a leadless pacemaker and a subcutaneous ICD.

## Case Reports

### Patient 1

A 52-year-old man with hypertension, obstructive sleep apnea, obesity, paroxysmal atrial fibrillation, and hypertrophic cardiomyopathy and a nonischemic cardiomyopathy with severe systolic dysfunction received cardiac resynchronization therapy defibrillator upgrade with atrioventricular node ablation for refractory rapid atrial fibrillation and recurrent inappropriate ICD shocks. This upgrade procedure was complicated by a pocket infection that progressed to bacteremia, and the system was extracted 2 months later, and replaced with a right-sided transvenous ICD system. After 4 months, the patient presented with recurrent *Staphylococcus aureus*. The system was extracted. It was decided to implant an LP and a subcutaneous ICD, given the patient’s recurring infections.

### Patient 2

A 39-year-old man with end-stage renal disease on hemodialysis via a right internal jugular catheter, nonischemic cardiomyopathy with severe systolic dysfunction, left ventricular assist device (LVAD) (HeartMate II, Abbott) implantation, and permanent atrial fibrillation with atrioventricular node ablation and cardiac resynchronization therapy defibrillator implantation presented to our institution with thinning followed by erosion of the skin over the inferior edge of the generator, leaving the lead and generator partially exposed and therefore infected. Blood cultures were negative. He ultimately underwent complete system extraction and same-day implantation of an S-ICD and LP in a similar fashion to patient 1. The alternate vector was chosen to minimize the chance of electromagnetic interference from the LVAD.^16^ Parameters have remained satisfactory over 5 months of follow-up.

## Discussion

**Figure 1:**
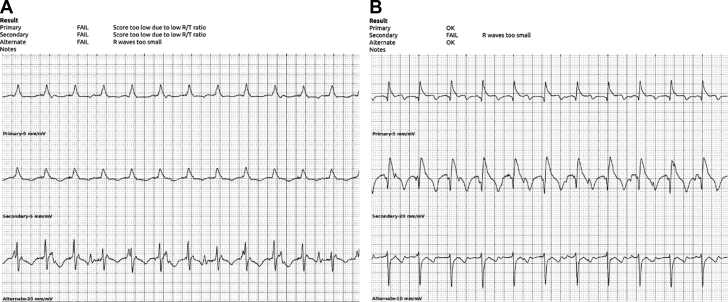
S-ICD Screening Electrograms During LP Pacing **(A)** Subcutaneous implantable cardioverter-defibrillator (S-ICD) waveform screening during pacing from the leadless pacemaker. At the initial site, screening failed in all vectors. **(B)** After repositioning, waveform screening passed in the primary and alternative vectors.
2) Redeployment of the LP to another site if screening fails. This is only possible if intraoperative mapping is performed.
3) Implantation of the S-ICD after the LP: this allows the lead to be implanted to the left or right of the sternum if needed, as dictated by the screening results.
4) Viewing live intracardiac electrograms from the LP during defibrillation efficacy testing to ensure that this device senses VF appropriately and does not pace during VF (Figure 2). To definitively test for this mode of crosstalk, the LP could be programmed VOO at high pacing output during one of the VF inductions, but with obligate bipolar pacing from the LP, this type of crosstalk is unlikely to occur.

Despite the use of preprocedural antibiotics, pocket irrigation, and use of antibiotic-eluting envelopes, CIED infections remain a major issue when they occur, with resultant morbidity and mortality.^1^ Here we report our workflow in performing simultaneous implantation of these devices. The key features are as follows:

1) Pacing from the LP at potential implant sites while performing screening for compatibility with the S-ICD (Figure 1). Electrode stickers are placed both left and right of the sternum, and the leads are manually switched under the drape by a nonsterile electrophysiology lab team member from left to right at each site screened.

**Figure 2:**
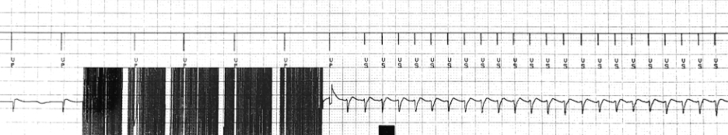
Intracardiac Electrogram From the Leadless Pacemaker During Defibrillation Efficacy Testing of the Subcutaneous Implantable Cardioverter-Defibrillator
5) There is a risk of inappropriate shocks due to electromagnetic interference oversensing in patients with an LVAD,^17^^,^^18^ but this can be minimized by programming sensing in the alternate vector.^19^ In our patient, we felt that the risk of bacteremia with an indwelling dialysis catheter, and the known elevated defibrillation threshold, made a transvenous system, implanted from the right pectoral or iliac routes, less favorable. Over follow-up, patient 2 has not received any inappropriate shocks.

## Conclusions

**Figure 3:**
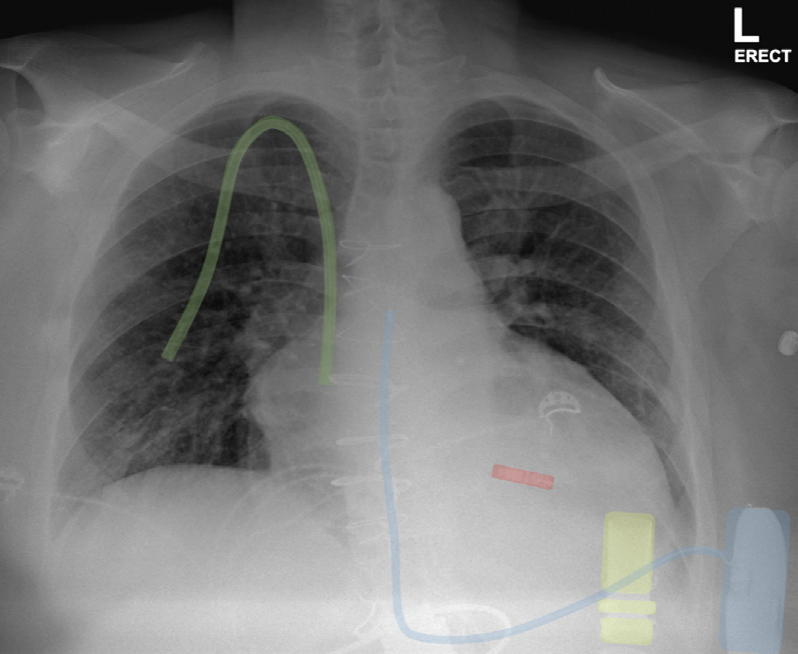
Anterior-Posterior View of the Leadless Pacemaker and S-ICD Systems Subcutaneous implantable cardioverter-defibrillator (S-ICD) system in **blue**, leadless pacemaker in **red**, right internal jugular catheter in **green**, left ventricular assist device cannula in **yellow**.

Our cases confirm that an LP can be implanted in conjunction with an S-ICD during a single procedure in patients who are unsuitable for the placement of a transvenous ICD system (Figure 3). This simultaneous implantation technique enables a workflow to look for device-device interaction and allows for corrective action to mitigate this interaction. This approach should be considered in patients who require both pacing and ICD functionalities, but in whom recurrent device infection or venous access limitations make transvenous lead implantation undesirable or impractical.

## Funding Support and Author Disclosures

Dr Cooper has received consulting fees from Abbott Medical, Johnson & Johnson, and Boston Scientific. All other authors have reported that they have no relationships relevant to the contents of this paper to disclose.
